# P62 inhibits IL-1β release during *Salmonella* Typhimurium infection of macrophages

**DOI:** 10.3389/fcimb.2025.1495567

**Published:** 2025-04-10

**Authors:** Nina Judith Hos, Julia Fischer, Ambika M. V. Murthy, Zahra Hejazi, Martin Krönke, Deniz Hos, Nirmal Robinson

**Affiliations:** ^1^ Institute for Medical Microbiology, Immunology and Hygiene, Faculty of Medicine and University Hospital Cologne, University of Cologne, Cologne, Germany; ^2^ Cluster of Excellence in Cellular Stress Responses in Aging-Associated Diseases (CECAD), University of Cologne, Cologne, Germany; ^3^ German Center for Infection Research (DZIF), Partner Site Bonn-Cologne, Cologne, Germany; ^4^ Department I of Internal Medicine, Division of Infectious Diseases, University of Cologne, Cologne, Germany; ^5^ Center for Molecular Medicine Cologne (CMMC), University of Cologne, Cologne, Germany; ^6^ Department B of Internal Medicine, University of Münster, University Hospital of Münster, Münster, Germany; ^7^ Center for Cancer Biology, University of South Australia and SA Pathology, Adelaide, SA, Australia; ^8^ Adelaide Medical School, Faculty of Health and Medical Sciences, The University of Adelaide, Adelaide, SA, Australia; ^9^ Department of Ophthalmology, Faculty of Medicine and University Hospital Cologne, University of Cologne, Cologne, Germany

**Keywords:** p62, ASC, IL-1β, IL-10, IFN-I, IFN-β, autophagy, inflammasome

## Abstract

Macrophages are critical for the innate immune defense against the facultative intracellular Gram-negative bacterium *Salmo\nella enterica* serovar Typhimurium. Following phagocytosis by macrophages, *S*. Typhimurium activates cytoplasmic NLRC3 and NLRP4 inflammasomes, which share the adaptor ASC, resulting in the secretion of the pro-inflammatory cytokine IL-1β. To prevent excessive inflammation and tissue damage, inflammatory signaling pathways are tightly controlled. Recently, autophagy has been suggested to limit inflammation by targeting activated inflammasomes for autophagic degradation. However, the importance of the autophagic adaptor Sequestome-1 (hereafter, p62) for regulating inflammasome activation remains poorly understood. We report here that p62 restricts inflammasome availability and subsequent IL-1β secretion in macrophages infected with *S*. Typhimurium by targeting the inflammasome adaptor ASC for autophagic degradation. Importantly, loss of p62 resulted in impaired autophagy and increased IL-1β secretion, as well as IL-10 and IFN-β release. In summary, our results demonstrate a novel role for p62 in inducing autophagy and balancing major pro- and anti-inflammatory signaling pathways to prevent excessive inflammation during *S*. Typhimurium infection of macrophages.

## Introduction

The Gram-negative bacterium *Salmonella enterica* serovar Typhimurium (*S*. Typhimurium) causes gastroenteritis in humans and typhoid fever in mice, which is accompanied by severe intestinal inflammation. After overcoming the intestinal epithelium, *S*. Typhimurium is targeted and phagocytosed by macrophages acting as a first line of defense against invading bacteria (Broz et al., 2012). Macrophages sense *S*. Typhimurium through their toll-like receptors (TLRs), which are activated by multiple pathogen-associated molecular pattern molecules (PAMPs), such as *S*. Typhimurium-derived lipopolysaccharide (LPS). LPS engages TLR4 signaling culminating in the activation of nuclear factor-κB (NF-κB) and interferon regulatory factor 3 (IRF3), which control several inflammatory cytokine cascades as well as type I interferon (IFN-I) production (Medzhitov, 2001). While proper activation of inflammatory signaling pathways is critical for innate and adaptive immune responses to *S*. Typhimurium, excessive inflammation causes tissue damage and systemic inflammatory response syndrome (SIRS) associated with increased morbidity and mortality (Ramachandran, 2014). Therefore, balancing pro-and anti-inflammatory responses is important to successfully combat infection.

Inflammasomes are intracellular multi-protein complexes that mediate the release of the pro-inflammatory cytokine interleukin (IL)-1β in response to a broad range of infectious stimuli (Schroder and Tschopp, 2010). Inflammasomes typically consist of a NOD-like receptor (NLR), which senses microbial or endogenous danger signals, and the adaptor Apoptosis-associated Speck-like protein containing a CARD (ASC), which is required for recruitment of pro-caspase-1 (Kelley et al., 2019). Following autocatalytic cleavage, caspase-1 proteolytically processes pro-IL-1β into its mature form, a process termed inflammasome maturation (Lamkanfi and Dixit, 2009). In macrophages, *S*. Typhimurium has been reported to synergistically activate NLRC3 (also known as Ipaf) and NLRP4 (also known as Nalp3) inflammasomes in a virulence-dependent manner (Broz et al., 2010). Inflammasome activation is critical for the innate immune defense against *S*. Typhimurium as mice lacking NLRC3 and NLRP4 or caspase-1 are more susceptible to *S*. Typhimurium infection (Broz et al., 2010).

Macroautophagy (hereafter, autophagy) has recently been suggested to restrict inflammation by targeting inflammasomes for lysosomal degradation (Shi et al., 2012). Autophagy is a highly conserved catabolic process by which protein aggregates or damaged cell organelles are engulfed into LC3 positive double-membrane vesicles, called ‘autophagosomes’. Autophagosomes subsequently fuse with lysosomes thereby promoting the proteolytic cleavage of engulfed cargo (Mizushima et al., 2008; Deretic et al., 2013). Proteins selected for autophagy are decorated with a ubiquitin signal that recruits the autophagic adaptor Sequestome-1 (hereafter, p62) (Matsumoto et al., 2011). P62 delivers ubiquitinated proteins for autophagic degradation by bridging ubiquitin chains and autophagosome-associated LC3 via its ubiquitin-associated (UBA) and LC3 interaction region (LIR), respectively (Pankiv et al., 2007; Matsumoto et al., 2011; Albornoz et al., 2019). Although p62 has extensively been studied during autophagy, its importance for regulating inflammasome activation remains poorly understood.

We report here that p62 restricts IL-1β release from *S*. Typhimurium-infected bone marrow-derived macrophages (BMDMs). Mechanistically, p62 was recruited to ASC-containing inflammasomes promoting their autophagic degradation. In addition to its negative impact on IL-1β secretion, p62 also impaired IL-10 and IFN-β release. Taken together, our findings identify p62 as a negative regulator of multiple inflammatory pathways and extend current knowledge on autophagy-dependent inflammasome regulation in macrophages.

## Results

### *S.* Typhimurium infection inhibits autophagy and promotes inflammasome activation

**Figure 1:**
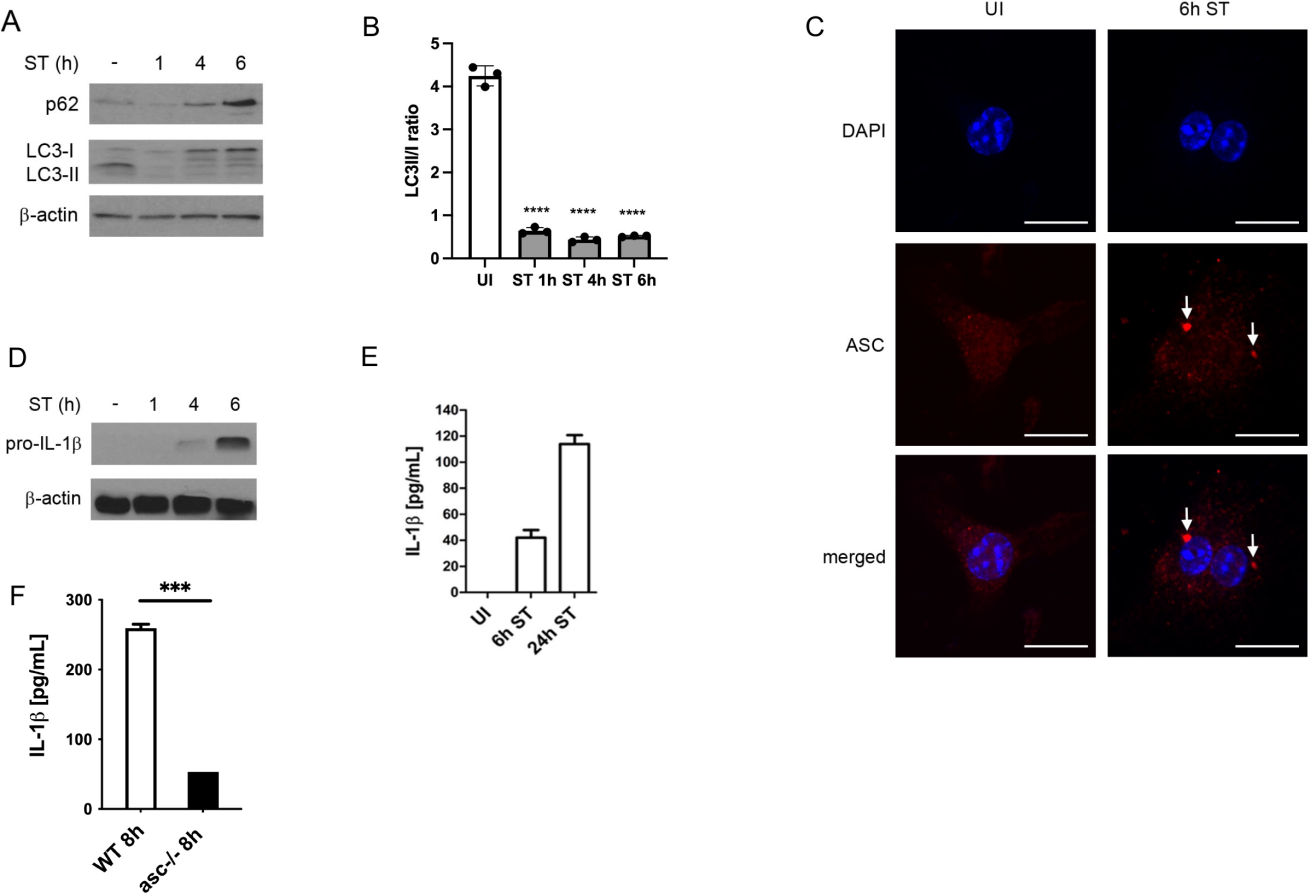
*S*. Typhimurium infection inhibits autophagy and promotes inflammasome activation. **(A)** Immunoblot analysis of autophagy marker proteins. BMDMs were infected with *S*. Typhimurium (ST) for the indicated time and p62 and LC3 expression was determined in total cell lysates by immunoblot. Upon autophagy induction, LC3-I is converted into lipidated LC3-II, which is recruited to the autophagosomal membrane. Both, p62 and LC3-II are degraded along with autophagic cargo and their expression levels are therefore inversely correlated with autophagic activity. **(B)** Quantification of LC3 II/I ratio by densitometric analysis **(C)** Immunofluorescence staining of activated inflammasomes. BMDMs were infected with *S*. Typhimurium (ST) for 6 h, immunostained for ASC (red) and examined by confocal microscopy (scale bars, 10 µm). Arrows point to ASC specks, indicative of activated inflammasomes. UI, uninfected. **(D)** Immunoblot analysis of IL-1β expression. Pro-IL-1β and caspase1 expression was determined by immunoblot in total cell lysates of BMDMs infected with *S*. Typhimurium (ST) for the indicated time. IL-1β is expressed as a pro-protein that requires caspase-1-mediated cleavage before secretion. **(E)** Analysis of IL-1β secretion by ELISA. BMDMs were infected with *S*. Typhimurium (ST) for 6 h and 24 h, respectively, and supernatants were collected and analyzed for IL-1β secretion by ELISA. Following cleavage of pro-IL-1β by caspase-1, mature IL-1β is secreted to the extracellular space. UI, uninfected. **(F)** Analysis of IL-1β secretion by ELISA. WT and ASC-/- BMDMs were infected with *S*. Typhimurium (ST) for 8 h and supernatants were collected and analyzed for IL-1β secretion by ELISA.

We first investigated the expression of the autophagic marker proteins p62 and LC3 following infection of BMDMs with *S*. Typhimurium. In line with our previous work (Ganesan et al., 2017; Hos et al., 2017), p62 levels were markedly decreased at 1 h of *S*. Typhimurium infection compared to uninfected BMDMs (**Figure 1A**). However, p62 expression was significantly enhanced after 4 h and 6 h of infection (**Figure 1A**). As p62 is degraded along with autophagic cargo, its protein levels are inversely correlated with autophagic activity. Accordingly, *S*. Typhimurium transiently induced autophagy at 1 h of infection, as assessed by enhanced LC3-I to LC3-II conversion rates (**Figure 1B**), which resulted in reduced p62 levels (**Figure 1A**). By contrast, LC3-I to LC3-II conversion was strongly reduced at 4 h and 6 h of infection (**Figure 1B**), indicative of autophagy inhibition, which promoted p62 accumulation (**Figure 1A**).

We next assessed *S*. Typhimurium-induced inflammasome activation. As displayed in **Figure 1C**, *S.* Typhimurium infection caused the formation of large ASC-positive protein complexes (>1 µm), termed ASC ‘specks’, which have been used as a marker for NLRC3 and NLRP4 inflammasome activation (Stutz et al., 1040). ASC is an essential component of NLRP3 and NLRP4 inflammasomes mediating the downstream activation of caspase-1, which cleaves pro-IL-1β into its active form that is subsequently secreted into the extracellular space (Lamkanfi and Dixit, 2009). Consistently, we found that pro-IL-1β was expressed in BMDMs after 4 h and 6 h of *S*. Typhimurium infection (**Figure 1D**). After 6 h and 24 h, mature IL-1β was secreted in the supernatants of *S*. Typhimurium-infected BMDMs (**Figure 1E**). Finally, IL-1 β was significantly reduced in ASC-knockout macrophages (**Figure 1F**).

These results demonstrate that *S*. Typhimurium inhibits autophagy whereas it promotes inflammasome activation.

### P62 restricts inflammasome availability

**Figure 2:**
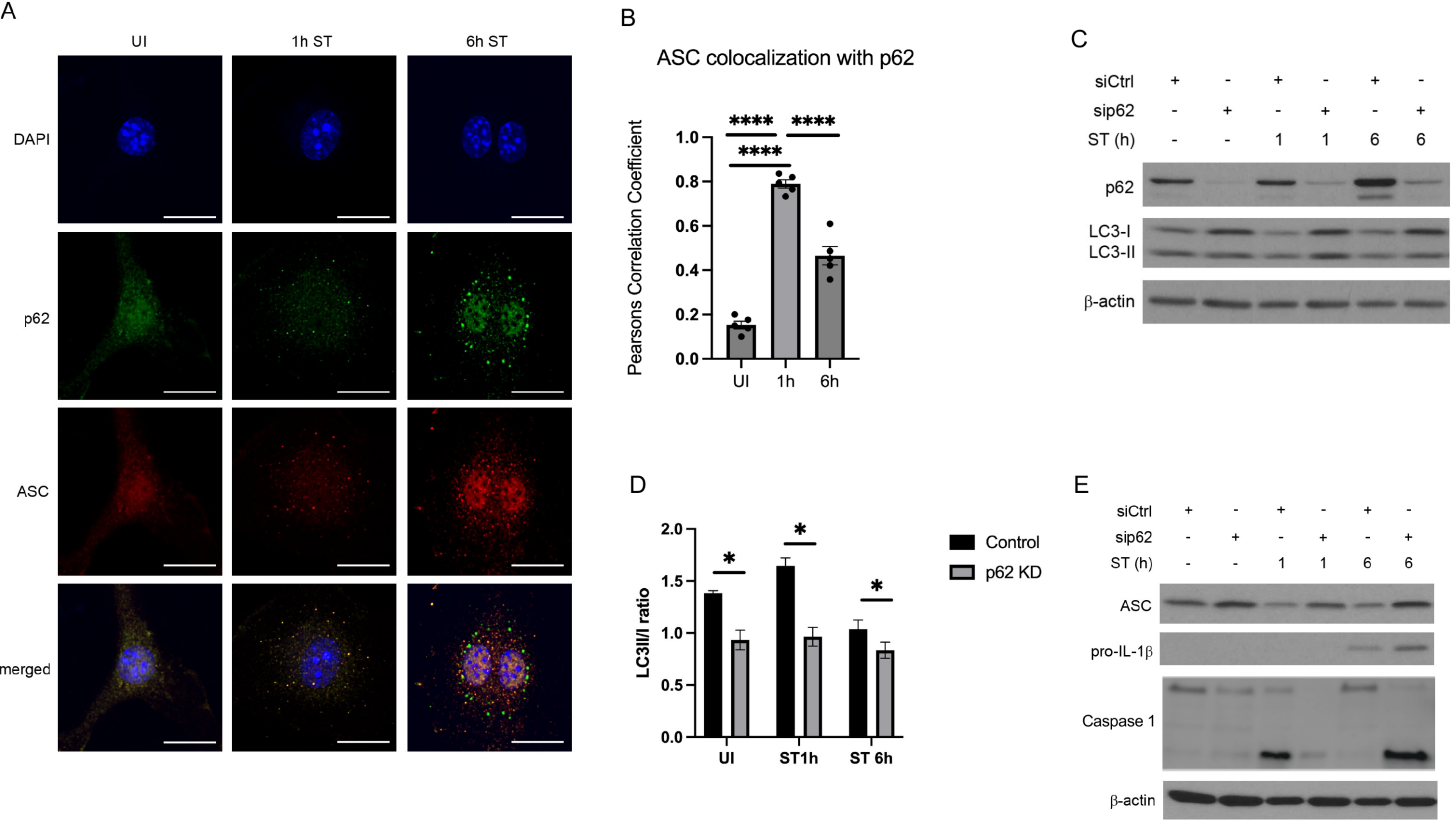
P62 restricts inflammasome availability. **(A)** Immunofluorescence staining of p62 and ASC co-localization. After 1h and 6 h of *S*. Typhimurium (ST) infection, BMDMs were immunostained for p62 (green) and ASC (red). P62 and ASC co-localization was examined by confocal microscopy. Scale bars, 10 µm. UI, uninfected. **(B)** quantification of colocalisation of asc and p62 analyzed with Person Correlation Coefficient **(C)** Immunoblot of p62-dependent autophagy initiation. BMDMs were transfected with non-targeting (siCtrl) or siRNA specific for *Sqstm1/p62* (sip62). At 48 h of transfection, BMDMs were infected with *S*. Typhimurium (ST) for the indicated time and p62 and LC3 expression in total cell lysates was determined by immunoblot. **(D)** Quantification of LC3 II/I ratio by densitometric analysis **(E)** Immunoblot of p62-dependent inflammasome availability. BMDMs were transfected with non-targeting (siCtrl) or siRNA specific for *Sqstm1/p62* (sip62) for 48 h. Thereafter, BMDMs were infected with *S*. Typhimurium (ST) for the indicated time and pro-IL-1β, caspase 1 and ASC expression was determined in total cell lysates by immunoblot.

Recent reports suggested that autophagy and inflammasome activation are tightly co-regulated. Following pharmacological inflammasome activation, ASC-positive inflammasomes have been shown to be targeted for autophagic degradation (Shi et al., 2012). To investigate if inflammasomes are also degraded by autophagy during *S*. Typhimurium infection, we analyzed whether the autophagic adaptor p62 is translocated to ASC-positive inflammasomes. As shown in **Figures 2A, B**, p62 strongly co-localized with ASC 1 h and 6 h after *S*. Typhimurium infection, indicating that ASC-containing inflammasomes were targeted for p62-mediated selective autophagy.

To assess the role of p62 in regulating autophagy and inflammasome activation in more detail, we transfected BMDMs with non-specific siRNA (siCtrl) or siRNA against *Sqstm1* (the gene encoding p62; sip62) prior to *S*. Typhimurium infection. In line with our previous finding (**Figure 1A**), LC3-I to LC3-II conversion, indicative of autophagy induction, was transiently triggered at 1 h of infection in siCtrl BMDMs (**Figure 2C**). After 4 h and 6 h of infection, LC3-I to LC3-II conversion was reduced, indicative of autophagy inhibition (**Figures 2C, D**). Importantly, knockdown of p62 resulted in reduced LC3-I to LC3-II conversion throughout infection (**Figure 2D**), demonstrating that p62 deficiency impaired autophagy initiation.

Given that p62 mediates selective autophagy of ASC-containing inflammasomes (**Figure 2A**), we hypothesized that genetic silencing of *Sqstm1/p62* results in ASC accumulation. Indeed, ASC expression was significantly increased in *S*. Typhimurium-infected sip62 BMDMs, which are defective in autophagy, compared to siCtrl BMDMs (**Figure 2E**). Likewise, pro-IL-1β expression and caspase 1 were also enhanced in *S*. Typhimurium-infected sip62 BMDMs compared to siCtrl BMDMs at 6h post infection when autophagy is inhibited and p62 accumulates (**Figure 2E**).

We therefore conclude that p62 inhibits inflammasome availability during *S*. Typhimurium infection by targeting major inflammasome components, ASC, caspase-1 and pro-IL-1β.

### P62 negatively regulates major inflammatory pathways

**Figure 3:**
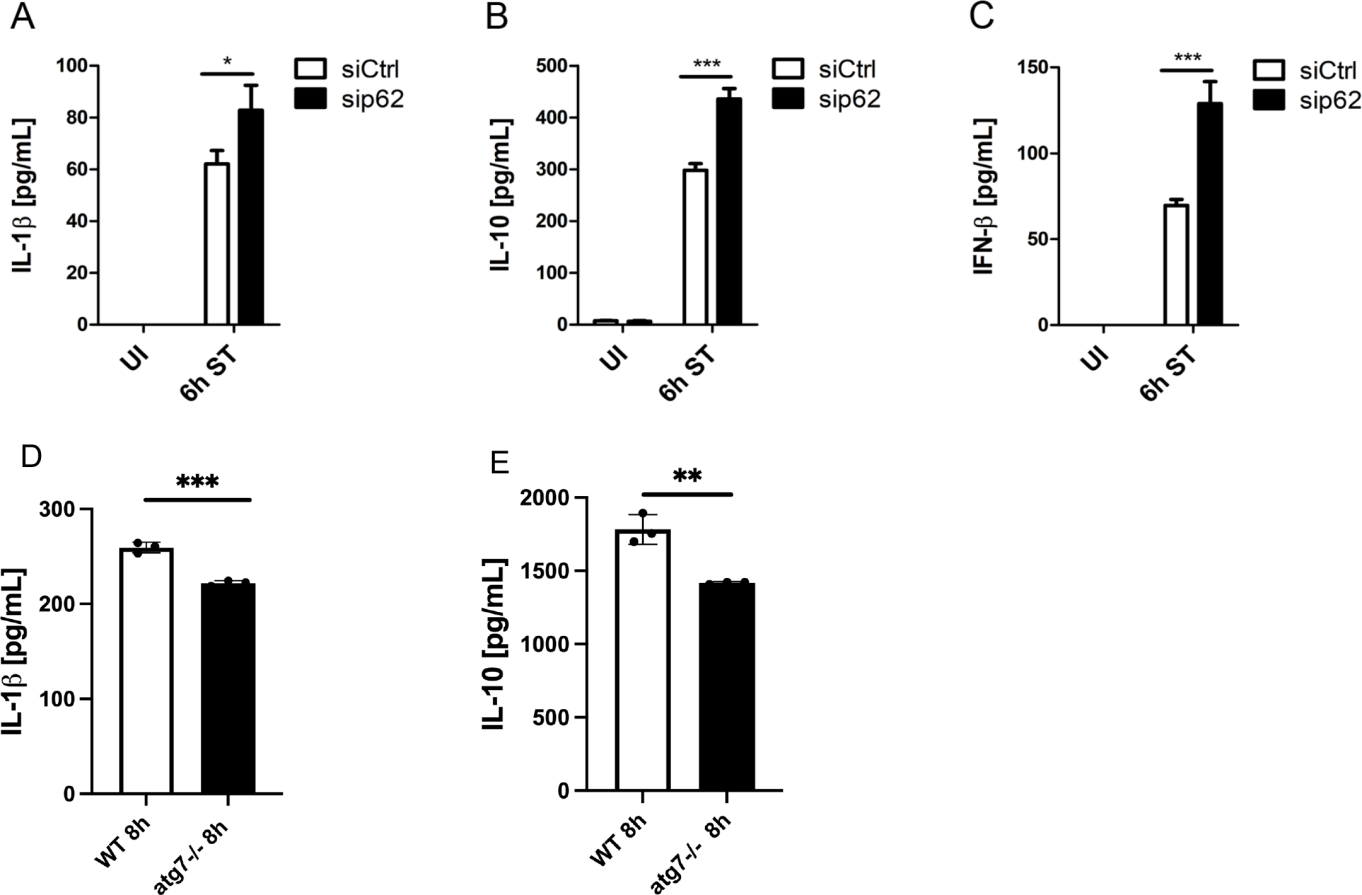
P62 negatively regulates major inflammatory pathways independent of autophagy. **(A–C)** P62-dependent IL-1β, IL-10 and IFN-β secretion. Following transfection with non-targeting (siCtrl) or siRNA specific for *Sqstm1/p62* (sip62) for 48 h, BMDMs were infected with *S*. Typhimurium (ST) for 6 **(h)** Thereafter, supernatants were collected and analyzed for **(A)** pro-inflammatory IL-1β, **(B)** anti-inflammatory IL-10 and **(C)** immunomodulatory IFN-β secretion by ELISA. UI, uninfected. **(D)** Analysis of IL-1β secretion by ELISA. WT and Atg7-/- BMDMs were infected with *S*. Typhimurium (ST) for 8 h and supernatants were collected and analyzed for IL-1β secretion by ELISA **(E)** Analysis of IL-10secretion by ELISA. WT and Atg7-/- BMDMs were infected with *S*. Typhimurium (ST) for 8 h and supernatants were collected and analyzed for IL-1β secretion by ELISA.

Having shown that p62 limits inflammasome availability through reducing ASC and pro-IL-1β levels, we next analyzed whether p62 also exerted a negative effect on inflammasome maturation. Compared to siCtrl BMDMs, knockdown of p62 significantly enhanced secretion of mature IL-1β after 6 h of *S*. Typhimurium infection (**Figure 3A**), indicating that p62 additionally impairs inflammasome maturation.

We next assessed the role of p62 in regulating anti-inflammatory signaling pathways. As shown in **Figures 3B, C**, *S.* Typhimurium induced the secretion of anti-inflammatory IL-10 and immunomodulatory IFN-β from siCtrl BMDMs 6 h after infection. Notably, *S*. Typhimurium-infected sip62 BMDMs secreted approximately 30% more IL-10 than the corresponding controls (**Figure 3B**). Furthermore, *S*. Typhimurium infection led to 2-fold higher IFN-β secretion from sip62 BMDMs compared to controls (**Figure 3C**). These results therefore suggest that p62 is a negative regulator of not only IL-1β but also other cytokines such as IL-10 and IFN-β during *S*. Typhimurium infection of macrophages.

Next, we asked if autophagy could restrict the increase in cytokine expression during *S*. Typhimurium infection. We found that IL-1β, IL-10 and were down regulated in Atg7-/- BMDMs infected with *S.* Typhimurium (**Figures 3D, E**). This suggests that increased cytokine secretion is independent of autophagy.

## Discussion

In our present work, we have investigated the role of the autophagic adaptor p62 in regulating inflammasome activation during *S*. Typhimurium infection of macrophages. We report here, that p62 impaired inflammasome activation and subsequent IL-1β secretion independent of autophagic degradation. Beyond restricting IL-1β release, p62 also decreased IL-10 and IFN-β secretion, indicating that p62 plays a critical role in balancing major pro- and anti-inflammatory signaling pathways during *S*. Typhimurium infection of macrophages.

We demonstrate here that p62 controls inflammasome availability to restrain IL-1β secretion from *S*. Typhimurium-infected macrophages. Specifically, we found that p62 was recruited to ASC. Accordingly, Shi et al. reported that p62 was translocated to ubiquitinated ASC on NLRP3 inflammasomes of THP-1-derived macrophages following treatment with LPS and ATP (Shi et al., 2012). While these findings confirm the importance of p62 for acting as an adaptor during selective autophagy of inflammasomes, our results additionally indicate that p62 plays a crucial role in promoting autophagy itself. Knockdown of p62 markedly reduced LC3-I to LC3-II conversion, indicative of impaired autophagy induction, resulting in the accumulation of the inflammasome components ASC and IL-1β. Our finding that p62 initiates autophagy is supported by a previous study showing that knockdown of p62 suppresses TLR4-mediated LC3 conversion in macrophages (Fujita et al., 2011). Together, these findings imply a role for p62 in regulating inflammasome pathway and autophagy during *S*. Typhimurium infection of macrophages.

Autophagy represents a major regulatory pathway for the control of activated inflammasomes in macrophages (Harris et al., 2011). Defective autophagy has been shown to promote IL-1β release in several inflammatory conditions, including *Mycobacterium tuberculosis* infection and Crohn´s disease (Saitoh et al., 2008; Kleinnijenhuis et al., 2011). In line with this notion, we demonstrate that blockade of autophagy upon knockdown of p62 increases IL-1β expression and secretion from *S*. Typhimurium-infected macrophages. Similarly, Zhong et al. reported that loss of p62 drives inflammasome activation and IL-1β secretion by impairing the autophagic removal of ROS-producing mitochondria from LPS-primed BMDMs treated with NLRP3 agonists (Zhong et al., 2016). Likewise, previous work from Sitoh et al. demonstrated that loss of the essential autophagy protein Atg16L1 impairs autophagy initiation and enhances IL-1β secretion from LPS-treated macrophages (Saitoh et al., 2008). Our findings at a first glance could suggest that autophagy negatively regulates inflammasome availability during *S*. Typhimurium infection of macrophages. However, autophagy has been also shown as a positive regulator of inflammasome activation and IL-1β secretion (Dupont et al., 2011). Furthermore, inflammasome also inhibits autophagy during infection (Suzuki et al., 2007). Thus, inflammasome activation and autophagy can regulate each other (Sun et al., 2017).

Our findings showing increased IL-1β secretion, IFN-β secretion and IL-10 upon loss of p62 and reduction of LC3 conversion could imply that reduced autophagy exacerbates inflammation. However, loss of autophagy during the later time points of infection reduces cytokine secretion. It is possible that accumulation of p62 due to inhibition of autophagy at later time points could be a compensatory mechanism employed by macrophages to limit inflammatory cytokine secretion. This is consistent with increased caspase-1 activation early time point (1h) when p62 levels are lower and autophagy is increased. Furthermore, previous studies have shown that p62-dependent P-bodies regulate inflammasome (Barrow et al., 2024). While our results clearly demonstrate a role for p62 in limiting pro-inflammatory IL-1β production, its importance for regulating anti-inflammatory immune responses remains controversial. Unlike inflammasome-dependent synthesis and secretion of pro-inflammatory IL-1β, are, the anti-inflammatory and immunomodulatory cytokines IL-10 and IFN-β, respectively, are secreted independent of caspase-1-mediated cleavage. In *S*. Typhimurium-infected macrophages, IFN-β is produced in response to TLR4 activation, which leads to nuclear translocation of IRF3 and transcription of *IFN-β*, the predominant IFN-I in mice (Barber, 2015). Upon binding to the transmembranic IFN-I receptor (Ifnar1), IFN-β promotes STAT1 and STAT2 dimerization as well as STAT3 homodimerization, resulting in the transcription of numerous IFN-I stimulated genes (ISGs) (Decker et al., 2005), including *IL-10* (Guarda et al., 2011). IL-10 has recently been reported to limit inflammasome availability through reducing pro-IL-1β expression in a STAT3-dependent manner (Guarda et al., 2011). Accordingly, our previous work demonstrated that IFN-β, which acts upstream of IL-10, decreases both IL-1β expression and secretion during *S.* Typhimurium infection of macrophages (Hos et al., 2017). Moreover, our present results indicate that IFN-β and IL-10 are additionally regulated by p62, because knockdown of p62 increased IFN-β and IL-10 secretion from *S*. Typhimurium-infected macrophages. It has recently been reported that autophagy limits poly I:C-induced as well as IFN-γ-mediated IFN-I expression by targeting IRF3 for autophagic degradation (Mathew et al., 2014; Kimura et al., 2017).

In summary, this study implies a novel mechanism by which macrophages restrict autophagy and enhance p62 to regulate pro- and anti-inflammatory immune responses during *S*. Typhimurium infection. While p62 is pivotal for controlling pro-inflammatory IL-1β levels, further studies are needed to investigate the significance of p62 for regulating anti-inflammatory pathways. Nonetheless, enhancing p62 function in macrophages may be a novel strategy to control excessive inflammation in various inflammatory disorders.

## Materials and methods

### Generation of bone marrow-derived macrophages

Bone marrow from 8 to 12 weeks old wildtype C57BL/6 (WT) mice was differentiated into macrophages in RPMI medium supplemented with 10% fetal bovine serum (FBS) and 20% L929 cell culture supernatant for 7 days. Non-adherent cells were removed on days 3 and 5. On day 6, cells were seeded into 6-well or 24-well sterile tissue culture plates, respectively, and adherent bone marrow-derived macrophages (BMDMs) were used for experiments from day 7 to 9. All animal procedures, including bone-marrow extraction from WT mice, were performed according to the institutional guidelines on animal welfare and were approved by the North Rhine-Westphalian State Agency for Nature, Environment, and Consumer Protection (Landesamt für Natur, Umwelt and Verbraucherschutz [LANUV] Nordrhein-Westfalen; File no: 84-02.05.40.14.082 and 84-02.04.2015.A443) and the animal care committee of the University of Cologne.

### *Salmonella* Typhimurium infection

*Salmonella enterica* serovar Typhimurium (SL1344) was grown to late-exponential phase in Brain Heart Infusion (BHI) broth, resuspended in 10% of sterile glycerol and stored at -80°C until further usage. BMDMs were infected with a multiplicity of infection (MOI) of 10 for 10 min at room temperature (RT) and 30 min at 37°C to allow bacterial invasion. BMDMs were washed with RPMI medium supplemented with 50 µg/mL gentamicin to remove extracellular bacteria and were incubated with RPMI medium containing 10% FBS and 50 μg/mL gentamicin at 37°C. Gentamicin was diluted to 10 µg/mL after 2 h of infection. At the indicated time points, BMDMs were washed once with PBS and samples were collected for experiments.

### Immunoblot analysis

After infection with *S*. Typhimurium for the indicated time, BMDMs (1.0-2.0 x 10^6^ cells/well) were lysed with radio-immunoprecipitation assay (RIPA) buffer containing protease and phosphatase inhibitors and stored at -20°C. Protein concentrations were estimated with the BCA Protein Assay Kit (Pierce, #23227) according to the manufacturer`s instructions. Equal amounts of protein were mixed with SDS-PAGE sample loading buffer, boiled for 10 min, resolved by SDS-PAGE and proteins were transferred to a PVDF membrane. After blocking with 5% milk diluted in tris buffered saline (TBS) containing 0.05% Tween 20 (TBS-Tween), membranes were incubated overnight at 4°C with one of the following antibodies: anti-ASC (Santa Cruz, #sc-22514), anti-IL-1β (R&D, #AF-401-NA), LC3 (Sigma Aldrich, #L7543), SQSTM1/p62 (Cell Signaling, #5114) or β-actin (Santa Cruz, #sc-47778). Next, membranes were treated with the appropriate secondary antibodies (R&D, #HAF008, #HAF007, # HAF109) for 1 h at RT, incubated with an enhanced chemiluminescence (ECL) substrate for 1 min and exposed to X-ray films that were automatically developed with the Curix60 machine (Agfa).

### Enzyme-linked immunosorbent assay

BMDMs (0.5-1.0 x 10^6^ cells/well) were seeded in 6-well plates and infected with *S*. Typhimurium for 6 h and 24 h. Thereafter, cell culture supernatants from three biological replicates were collected and stored at -80°C until assayed for IL-1β (R&D, #DY401), IL-10 (R&D, #DY417) or IFN-β (pbl, #42410-1) according to manufacturer´s instructions. Absorbance was measured with a PerkinElmer multimode plate reader at 450 nm.

### Immunofluorescent staining and confocal microscopy

BMDMs (0.1 x 10^6^ cells/well) seeded in 24-well plates containing 12 mm coverslips were infected with *S*. Typhimurium. At the indicated timepoints, BMDMs were fixed with 4% (wt/vol) formaldehyde in PBS for 15 min and permeabilized with 0.3% Triton X-100 in PBS for 5 min. Coverslips were treated with Image-iT^®^ R FX signal enhancer (Invitrogen, # I36933) for 30 min and blocked with 10% BSA diluted in 0.03% Triton X-100 in PBS for 1 h at RT. Next, BMDMs were incubated overnight at 4°C with primary antibodies against SQSTM1/p62 (Cell Signaling, #5114) and ASC (Santa Cruz, #sc-22514) and appropriate fluorescent secondary antibodies (Invitrogen, #A-11008, #A-11072) for 1 h at RT. Coverslips were mounted on glass slides using ProLong^®^ Gold antifade containing DAPI (Cell Signaling, #8961) and stored at 4°C until image acquisition. Images were acquired with a 60X oil PlanApo objective on an Olympus IX81 inverted confocal microscope. Olympus Fluoview FV10-ASW 4.2 software was used for image acquisition.

### Transfection experiments

BMDMs (1.0 x 10^6^ cells/well) were incubated with either 50 nM of non-targeting siRNA (Eurogentec, #SR-CL000-005) or 50 nM of siRNA specific for *Sqstm1/p62* (Dharmacon, #L-047628-01-0005) together with the transfection reagent Lipofectamine 3000 (Thermo Fisher, #L3000-008) according to the manufacturer’s guidelines. At 48 h post transfection, BMDMs were infected with *S*. Typhimurium for the indicated time and total cell lysates and supernatants were collected for further experiments.

### Statistical analyses

Statistical analyses were performed using Prism software (GraphPad, version 5). Differences between groups were assessed by two-tailed unpaired Student´s t test. Data are representative of at least three independent experiments and results are presented as mean ± standard deviation (SD). Differences were considered statistically significant when p ≤ 0.05 (∗), very significant when p ≤ 0.01 (∗∗), and highly significant when p ≤ 0.001 (∗∗∗).

## Data Availability

Requests to access the datasets should be directed to hosnina@yahoo.de.
